# An analysis of the gene interaction networks identifying the role of PARP1 in metastasis of non-small cell lung cancer

**DOI:** 10.18632/oncotarget.20256

**Published:** 2017-08-14

**Authors:** Kai Chen, Yajie Li, Hui Xu, Chunfeng Zhang, Zhiqiang Li, Wei Wang, Baofeng Wang

**Affiliations:** ^1^ Department of Respiratory Medicine, Baoji Central Hospital, Baoji 721008, Shaanxi, China; ^2^ Department of Cardiology, Baoji Central Hospital, Baoji 721008, Shaanxi, China

**Keywords:** NSCLC, gene interaction network, shortest path, PARP1, migration and invasion

## Abstract

**Background and Objective:**

Though there were many researches about the effects of cancer cells on non-small cell lung cancer (NSCLC) currently, it has been rarely reported completed oncogene and its mechanism in tumors by far. Here, we used biological methods with known oncogene of NSCLC to find new oncogene and explore its functionary mechanism in NSCLC.

**Methods:**

The study firstly built NSCLC genetic interaction network based on bioinformatics methods and then combined shortest path algorithm with significance test to confirmed core genes that were closely involved with given genes; real-time qPCR was conducted to detect expression levels between patients with NSCLC and normal people; additionally, detection of PARP1's role in migration and invasion was performed by trans-well assays and wound-healing.

**Results:**

Through gene interaction network, it was found that, core genes like PARP1, EGFR and ALK had a direct interaction. TCGA database showed that PARP1 presented strong expression in NSCLC and the expression level of metastatic NSCLC was significantly higher than that of non-metastatic NSCLC. Cell migration of NSCLC in accordance to the scratch test was suppressed by PARP1 silence but stimulated noticeably by PARP1 overexpression. According to Kaplan-meier survival curve, the higher PARP1 expression, the poorer patient survival rate and prognosis. Thus, PARP1 expression had a negative correction with patient survival rate and prognosis.

**Conclusion:**

New oncogene PARP1 was found from known NSCLC oncogene in terms of gene interaction network, demonstrating PARP1's impact on NSCLC cell migration.

## INTRODUCTION

Non-small cell lung cancer (NSCLC), a leading neoplastic disease [1] with the highest incidence rate and cancer-related mortality worldwide, has a 5-year survival rate under 10% due to high metastasis rate and poor prognosis. Resection has been the main therapy by far [2].

Targeted therapy for oncogene has been an emerging therapeutic method in recent years, whereas patient survival rate has not been improved due to late detection and therapy. Thus it was essential to find cancer marker guidance of early discovery, diagnosis and treatment, which led to biomarker becoming one of the most valuable area in cancer research. NSCLC is hardly characterized by one marker for heterogeneous disorder with a variety of biomarker [3], making it hard to find effective NSCLC marker.

With rapid development of high-throughput biotechnology, biological data have been obtained from protein complex and gene expression profile, which helped to clarifying gene function and resource acquisition. Some scholars observed differentially expressed genes with microarray between Lung adenocarcinoma and adjacent normal tissue and established protein-protein interaction (PPI) network with gene ontology (GO) and Kyoto encyclopedia of genes and genomes (KEGG) for enhancement of lung adenocarcinoma knowledge [4]. PPI search tool of network construction and interaction gene STRING database provided mutual information for experiment and prediction. To get gene-related information, studying topological features of disease gene product in PPI network has become a new way of gene predicting.

Closely related core genes could be found by means of gene interaction network starting with known NSCLC gene. PARP1 had a high connectivity, namely as a basic core, and directly attracted with typical oncogene EGFR and ALK; mutating EGFR in NSCLC constitutively activated encoding protein and caused the occurrence of abnormal function and constant signal transduction out of control, which was in correction with NSCLC development [5]; as a representative molecularly targeted agent, ALK inhibitor could activate recombination through mutation, gene amplification and chromosome abnormality to increase carcinogenic driver expression [6].

Furthermore, TCGA indicated that PARP1 was highly expressed in NSCLC, suggesting that PARP1 had a great influence on NSCLC. Coded protein in eukaryotic cells of PARP1 catalyzes poly-ADP-ribosylation and is involved in the process of DNA single-strand damage repair. The main achievement of PARP1 in cancer is stimulation for proliferation of colorectal cancerous cells, whereas there is no research on PARP1 functional mechanism in NSCLC.

The study firstly surveyed PARP1's effect on NSCLC cells and discussed its functionary mechanism, which found that PARP1 was expressed strongly in metastatic NSCLC; and high PARP1 expression was relevant to prognosis and facilitated NSCLC cells migration and invasion, which provided not only corresponding foundation of experiment medicine for later targeted treatment studies but theory support of PARP1 expression within NSCLC and possible mechanism research.

## RESULTS

### The known genes of non-small cell lung cancer

OMIM (Online Mendelian Inheritance in Man) is a comprehensive database involved in human gene and genetic disease [7]. There were 3358 annotation of genes related to mutation phenotype of Mendelian inherited disease by March, 16^th^, 2015. Seven genes were identified from the search with the key word “non-small cell lung cancer”.

The database COSMIC (Catalogue of Somatic Mutations in Cancer) [8] embodied cancer-related gene extracted in high-throughput experimental data which was from the reported papers and cancer genome plan of Sanger laboratory. 19 genes were identified from the search with the key word “non-small cell lung cancer” in this database.

GAD (The Genetic Association Database) [9] is a comprehensive database collecting complex disease of human. The reported literature and pathogenic gene of complex disease was annotated in GWAS experimental data. 12 genes were identified from the search with the key word “non-small cell lung cancer” in this database.

The database DisGeNET [10] annotate pathogenic gene by integrating common database and gene-disease relationship in reported literature. Currently 381,056 gene-disease relationships included in this database (including 16,666 genes and 13,172 kinds of diseases). There are 39 genes were identified from the search with the key word “non-small cell lung cancer” in this database. In summary, 62 genes were identified through searching database in total (no repetition). The specific gene name and database source were included in the Supplementary Table 1.

Though many databases were developed to collect pathogenic gene, there were some differences in the data with disease phenotype covered and various orientation of genotype due to the data types of different database. Meanwhile, the collection of information from above database might be incomplete considering the lag characteristic in the maintaining process of database.

Thus, there was a need for pathogenic gene through artificial screening to further analyze in terms of specific disease phenotype. Detection of the relationship between gene and non-small cell lung cancer conducted in Pubmed, gene name and non-small cell lung cancer as the searched key words, that is (“gene symbol” or “gene”) and “non-small cell lung cancer”. The literature would be recorded if the two key words appeared both in title and abstract of the same literature, as the evidence of detection of the association between gene and non-small cell lung cancer. Finally, 81 pathogenic genes were gained from the literature, specific gene name included in the Supplementary Table 1.

### Shortest path analysis of genes

**Figure 1:**
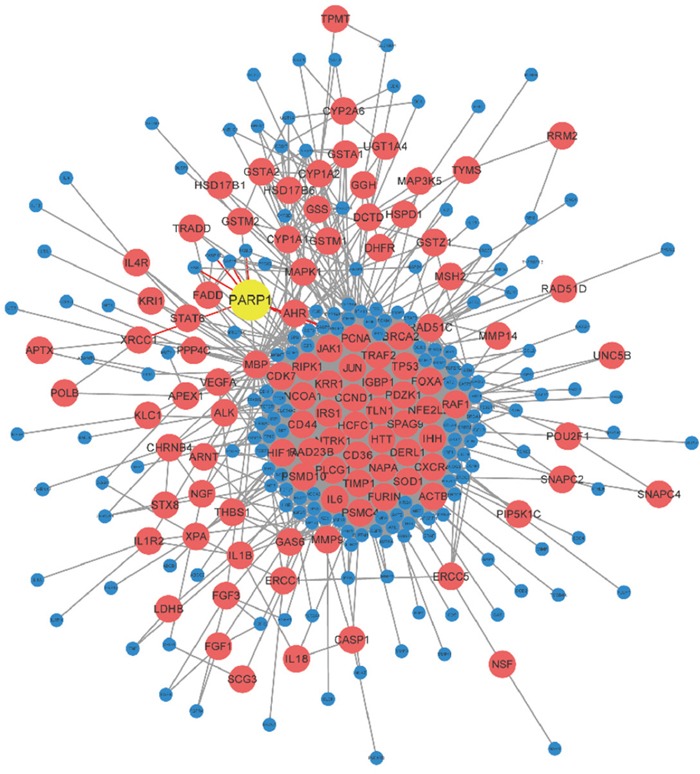
Network figure of NSCLC genes The PPI data of NSCLC was gained from STRING, and the connection of their shortest path was calculated through Dijkstra's algorithm. It indicated that PARP1 involved in the occurrence and development of NSCLC.

A non-directional network chart with the PPI data gained from STRING was established, and the connection of their shortest path through Dijkstra's algorithm was calculated (Figure 1). Then the Betweenness value of internal node in these paths was counted. 385 genes whose Betweenness value over 0 were gained, and these genes were listed out in the Supplementary Table 2. Permutation test was employed to check and calculate their permutation FDR in terms of further selection of these genes, similarly the results showed in the Supplementary Table 2.

Among them, permutation FDR of 103 genes were less than 0.05. There was a strong correlation between these genes and NSCLC, which could be used in the subsequent analysis. A gene PARP1 with high betweenness value except for the famous oncogene with high connected degree was found, which indicated it played at a relatively core role in NSCLC gene network, yet it was unknown of the role of this gene in lung cancer.

There was direct gene connections between PARP1 and the famous NSCLC genes, EGFR and ALK. Thus it was possible that PARP1 involved in the occurrence and development of NSCLC. Therefore, functional experiment was further adopted to detect the effect of PARP1 on NSCLC.

### The high expression of PARP1 in non-small cell lung cancer and metastatic non-small cell lung cancer

**Figure 2:**
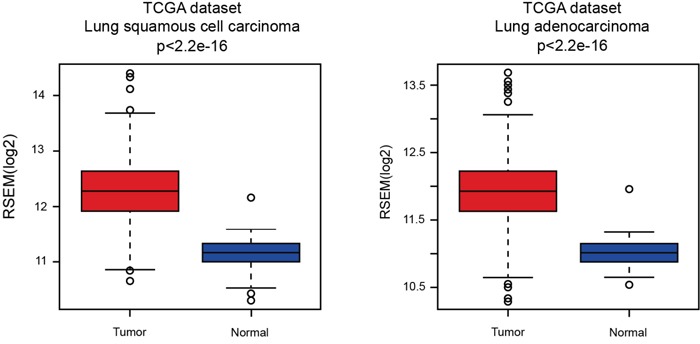
Expression of PARP1 in lung cancer The expression of PARP1 of squamous cell carcinoma (left) and adenocarcinoma (right) was gained from TCGA. The data of squamous cell carcinoma and adenocarcinoma in TCGA indicated significant higher expression of PARP1 in tumor tissues.

The expression of PARP1 in lung cancer with the expression data of TCGA was detected firstly. The data of squamous cell carcinoma and adenocarcinoma in TCGA indicated significant higher expression of PARP1 in tumor tissues (Figure 2).

Next the detection was conducted in the 75 patients with NSCLC disease. Among the 75 patients (40 patients of non-metastatic NSCLC and 35 patients of metastatic NSCLC) no differences was shown in their age, sex and smoking status.

**Figure 3:**
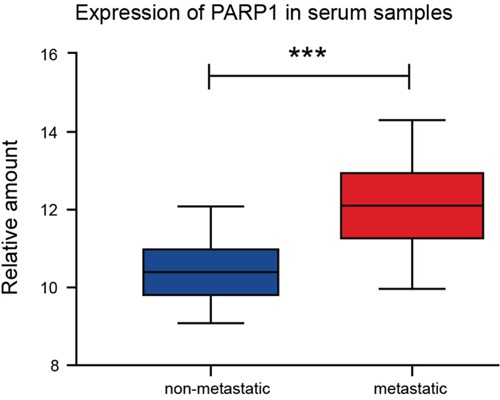
Higher expression of PARP1 in the serum samples ofmetastatic-NSCLC patients Q-PCR was used to detect the PARP1 expression of serum samples of 75 NSCLC patients. The results were analyzed using t-test. ^*^p < 0.05; ^**^p < 0.01; ^***^p < 0.001. Red, metastatic group; blue, non-metastatic group.

The PARP1 expression in serum samples was done by q-PCR, which indicated that the expression of PARP1 in metastatic NSCLC was significantly higher than that in non-metastatic NSCLC in blood samples (Figure 3).

### The high expression of PARP1 in metastatic non-small cell lung cancer

**Figure 4:**
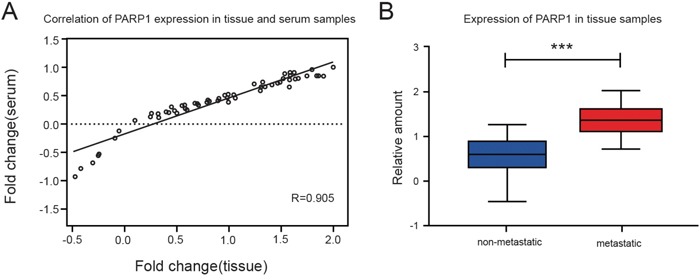
The expression of PARP1 in the tissue samples of NSCLC patients Q-PCR was used to detect the PARP1 expression of tissue samples of 75 NSCLC patients. **(A)** The PARP1 expression of Pearson correlation scatter diagram in the matching tissue of 75 NSCLC patients and serum samples. A high consistency was found in its expression of blood and tissue samples. **(B)** The expression of PARP1 in metastatic NSCLC was higher than that in the non-metastatic NSCLC tissue. The results were analyzed using t-test. ^*^p < 0.05; ^**^p < 0.01; ^***^p < 0.001. Red, metastatic group; blue, non-metastatic group.

We explored the expression of PARP1 in blood samples and tissue samples of NSCLC which showed a high consistency in its expression of blood and tissue samples (Figure 4A). It was indicated that the expression of PARP1 in blood could instruct the expression in tissue.

The results indicated that the expression of PARP1 in metastatic NSCLC tissues was higher (columnar section) than that in metastatic NSCLC tissue and non-metastatic NSCLC tissue (Figure 4B).

### Efficiency of PARP1 silence and overexpression in NSCLC cell line

A549, H1975 and PC9 were chosen to represent the typical NSCLC cell line in this study. Function analysis was conducted *in vitro* cell line to study the effects of PARP1 on NSCLC invasion by adopting overexpression and inhibition of PARP1 expression. The overexpression and silence cell line of PARP1 gene with NSCLC cell line A549, H1975 and PC9 was established, then the PARP1 expression was detected in the 3 cell lines.

**Figure 5:**
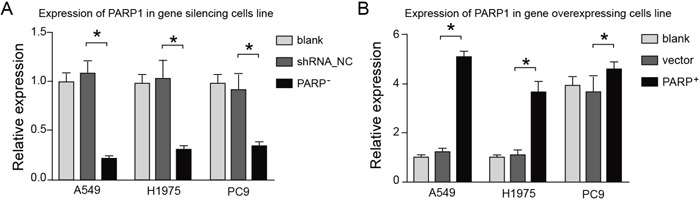
The effects of overexpressing or silencing plasmids on the PARP1 expression The expression of PARP1 in silence cell line decreased 70-80% compared with that of the control group (left), whereas the expression of PARP1 in overexpression cell line increased significantly (right).

The q-PCR results showed that the expression of PARP1 in silence cell line decreased 70-80% compared with that of the control group, and the expression of PARP1 in overexpression cell line increased (Figure 5).

### Promoting effect of PARP1 on proliferation, migration and invasion of NSCLC cell

Higher expression of *PARP1* was showed in NSCLC patients and metastatic NSCLC patients in the above study, which indicated *PARP1* involved in the metastatic process of NSCLC. Thus, PARP1 was necessary for the cell proliferation, migration and invasion during the metastatic process.

**Figure 6:**
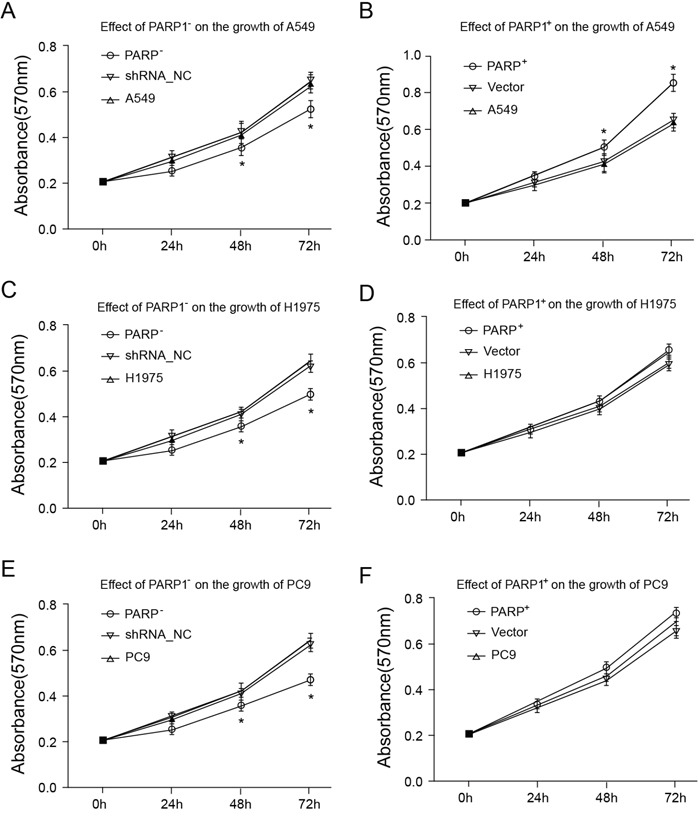
Cell proliferation was assessed with an MTT assay 2 × 10^4^/ml cell suspension fluid was inoculated in 96-well plates, and cultured for 96 h, respectively. Following that, the plates were added with MTT solution, and incubated for 4 h, then added with DMSO to fully resolve MTT pyrolysis products. Optical density (OD) was measured by immunoassay analyzer at the wavelength of 570 nm. **(A, B)** Effect of PARP1 on the growth of A549; **(C, D)** effect of PARP1 on the growth of H1975; **(E, F)** effect of PARP1 on the growth of PC9.

**Figure 7:**
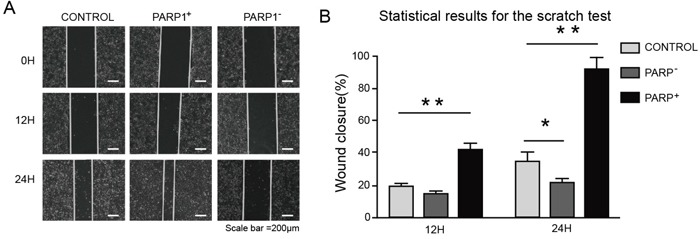
Promoting effect of PARP1 on migration of NSCLC cell For the scratch test, cells were plated at 2×10^5^ cells/well in a 6-well plate and grown overnight under standard conditions. After 12 h, the confluent monolayer was scratched manually with a plastic 200 μl pipette tip and after washing with PBS. Then the culture was continued in 2% serum at 37°C for 24 h. The wound area were imaged using an inverted microscope at 100× magnification. The distance was measured using ImageJ2x. **(A)** Images of wounds of A549 cells transfected with the indicated plasmids taken 0, 12 and 24 h afer the wounds were inflicted (× 100). **(B)** Overexpressing PARP1 promoted the wound-healing process in A549 cells, whereas PARP1 silencing did not contribute to wound healing. Wound-healing results for A549 cells transfected with the indicated plasmids (analyzed by t-tests). ^*^p < 0.05; ^**^p < 0.01;^***^p < 0.001.

**Figure 8:**
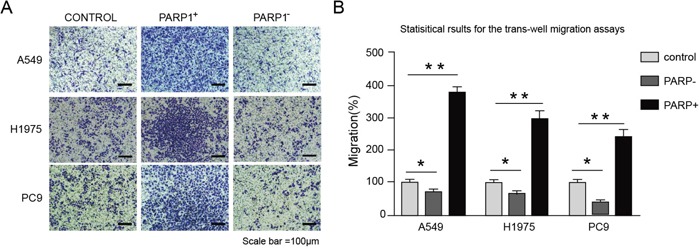
Overexpressing PARP1 promotes the metastasis of NSCLC cells lines, whereas PARP1 silencing inhibited the processes 1 × 10^5^ cells were plated onto 24-well cell chambers (pore size: 8 μm). The cells were plated in medium without serum, and medium with 20% serum was used as a chemoattractant. The cells were incubated at 37°C for 16 h. Cells in 10 random fields of view at 100× magnification were counted. Images were acquired using an inverted phase-contrast microscope at 100× magnification. **(A)** Images from the Trans-well migration assays of A549, H1975 and PC9 cells transfected with indicated plasmids (× 100). **(B)** Statistical results for the trans-well migration assays of A549, H1975 and PC9 cells transfected with indicated plasmids (analyzed by t-tests); %migration = [mean number of cells invading the membrane/mean number of cells migrating through the control insert membrane] × 100. ^*^p < 0.05; ^**^p < 0.01; ^***^p < 0.001.

Thus, firstly the effects of *PARP1* on the cell proliferation of A549, H1975 and PC9 were tested through MTT assays. The result indicated that *PARP1* gene silence could significantly inhibit the cell growth of A549, H1975 and PC9, while *PARP1* overexpression promoted the growth of A549 cell significantly (Figure 6). Secondly, cell scratch test would be conducted to identify whether *PARP1* could promote the metastatic capacity of NSCLC. The result showed that *PARP1* silence inhibited cell migration significantly shown in cell scratch test, while PARP1 overexpression promoted migration of NSCLC cell significantly (Figure 7). Finally, trans-well assays (Figure 8) was adopted to confirm these results, with the same conclusion drawn.

### PARP1 as prognostic gene

**Figure 9:**
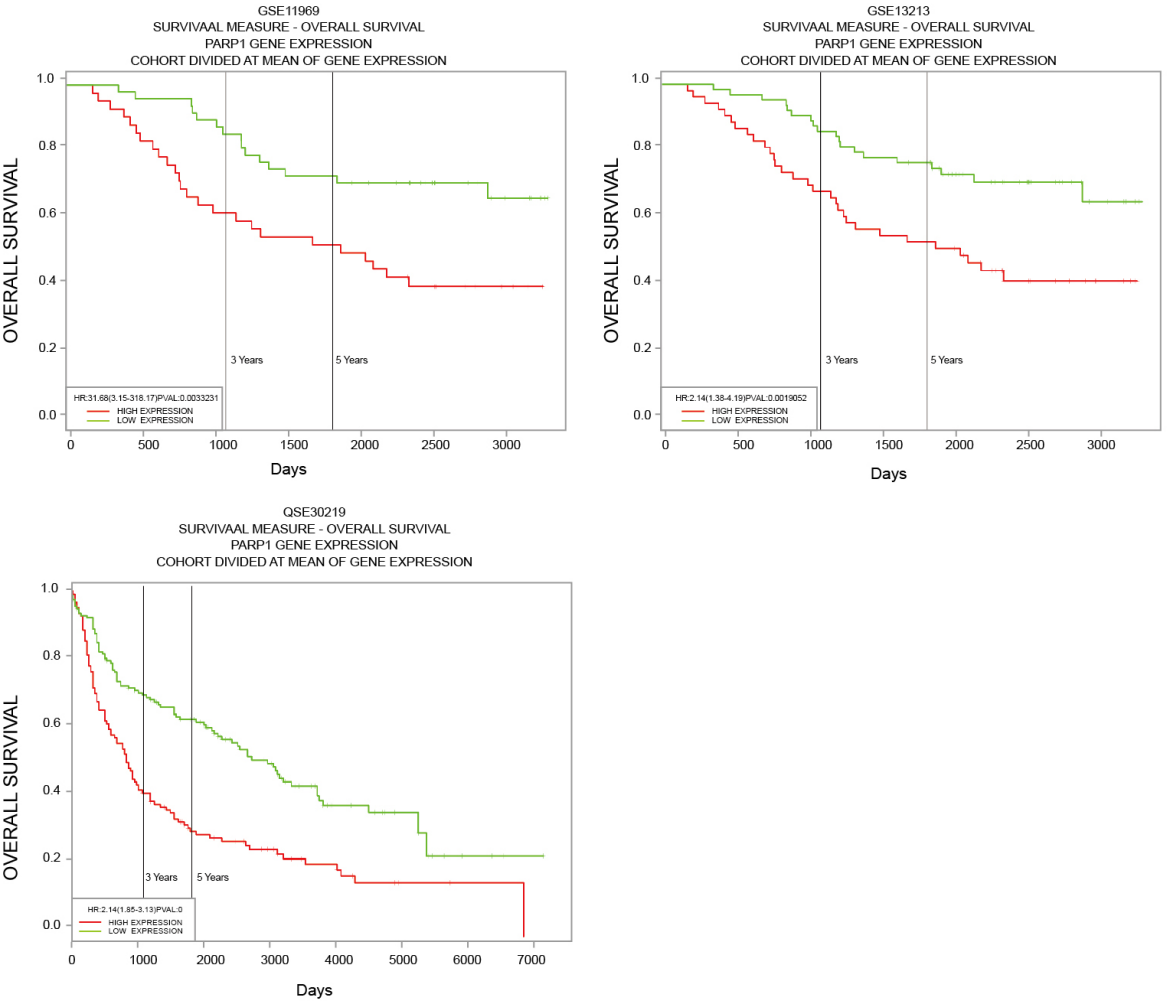
Survival relationship between PARP1 and patients of NSCLC Data from GSE11969, 13213 and 30219 was gained from GEO database, then relationship between expression of PARP1 and the prognosis or recurrence of patients was tested through Kaplan-Meier survival curve using the online survival analysis tool, PROGgene.

The survival relationship between PARP1 and patients of NSCLC was analyzed with the data in GEO database. Kaplan-meier survival curve was made using the online survival analysis tool, PROGgene. It was found that the expression of PARP1 was related to the prognosis or recurrence of patients through Kaplan-meier survival curve: The overall survival (OS) in PARP1 high expression group was significantly lower than that in PARP1 low expression group (Figure 9).

## DISCUSSION

Complex diseases were generally affected by the interaction of multiple genes [11]. Generally, the common influence of interaction in interacting genes could not only decrease the complex degree of biological network, but also benefit to explore meaningful biological information for the researchers, which further provide scientific basis for therapy and study of disease. Previous studies indicated that many interacting genes had similar or same effects, or involved in the same biochemical pathways. Therefore, the known key gene in an established interaction network of genes would be found easily, and the functions of interacting genes can be preliminary predicted and annotated based on this gene. In this study, NSCLC-related gene was searched from database OMIM, COSMIC, GAD and DisGeNET, then a network chart was established with the adoption of PPI data from STRING, followed by the shortest path analysis and Permutation test. There is a total of 385 genes whose Betweenness value was greater than 0 gained through Dijkstra's algorithm. Permutation test showed 103 genes whose Permutation FDR was less than 0.05. There was a strong correlation between these genes and NSCLC, which could be used in the subsequent analysis.

Almost all the NSCLC patients would recurred in different stages, and finally suffered from disease metastasis and death [12]. Even though there was a new development of immunotherapy and targeted therapy of tumors, once the systematic metastasis of cancer cell occurred, the five-year survival rate would decrease at lessby 10% [13]. For that it was still a challenge to expand the survival rate of NSCLC patients by inhibiting the cell migration of cancer cells. Therefore, it was of significance for the study of molecular markers and potential therapeutic small molecules in clinical stage. PARP1 played an important role in DNA damage repair, especially in base excision repair (BER) pathway [14]. It was further verified that PARP1 played a key role in DNA repair [15–17], which made PARP1 a valuable therapeutic target in the research of disease drug. Recently, it was found that PARP1 presented high expression in a series of cancers, which provided a high-value target for the study of tumor detection, stage and biological characteristics [18–23]. The overexpression of PARP1 results from much DNA damage in cancer cells with unstable genes, instead of the induction of the activated specific carcinogenic pathway [24]. Byers, et al. demonstrated that PARP1 could be a new target molecule for NSCLC by combined transcriptome and proteome analysis [25]. Meanwhile, a new study claimed that PARP1 could promote the metastasis and recurrence of lung adenocarcinoma towards brain and bones by regulating several steps in the metastasis process, which was not related to DNA repair. There were several ways of PARP1 enhancing the metastasis of lung adenocarcinoma towards brain: promoting the invasion of lung adenocarcinoma cells, anoikis resistance, exosmosis, and self-updating, as well as regulating cerebral microenvironment [26].

In the current study, the expression of PARP1 in the blood samples showed a high consistency with that in the tissue samples, which indicated that the expression of PARP1 in blood could instruct the expression in tissue. Because of that, the related information about the development of tumor could be obtained through the detection of PARP1 expression in blood at the early stage of NSCLC tumor. The results of MTT assays indicated that lockdown of PARP1 gene significantly inhibited the cell growth of A549, H1975 and PC9, while PARP1 overexpression promoted the growth of A431 cell significantly. It indicated that PARP1 gene promoted the proliferation of NSCLC, whose role was consistent in others malignant tumor [27, 28]. There were many extensive researches on the gene which influence NSCLC cell migration, mainly including MALAT-1 [29], MTA1 [30], BRAF [31], EGFR and so on. However, the role of PARP1 in NSCLC migration remains still unknown. Our experiments revealed that: expression of PARP1 was upregulated in NSCLC and showed higher expression in metastatic NSCLC; PARP1 silence inhibited cell migration significantly, while PARP1 overexpression promoted NSCLC cell migration significantly [26]. It has demonstrated that knockout or inhibition of PARP1 could decrease the migration of lung adenocarcinoma significantly, which indicated PARP1 was involved in the migration of NSCLC. In addition, there was a direct association between PARP1 and the key cancer gene of NSCLC: EGFR, ALK in the interaction network of genes. Previous studies have suggested that EGFR takes part in DNA repair through PI3K signaling pathway [32–33]. In the cells with ALK fusion, it has illustrated that dimers can be activated abnormally by aberrant receptors, which can activate the downstream signaling pathway PI3K-AKT and drive aberrant proliferation of tumor cells [34–35]. Based on these findings, it can be see that ALK and EGFR after requiring functional mutation could drive the growth of tumor cells via PI3K-AKT pathway. So we hypothesized that the effects of PARP1 on NSCLC tumor migration may be associated with the PI3K-AKT pathway. However, the mechanism needs further exploration.

It is of directive significance to understand the effect of prognostic factors on the prediction, diagnosis, and treatment of diseases. According to the study, PARP1 may be an independent prognostic factor of multiple malignant tumors. And PARP1 Val762Ala polymorphism may be an independent prognostic factor for cervical cancer, which might play a role in the prediction of clinical results [36]. Collectively, the expression of PARP1 might be considered as an independent prognostic factor for the patients of breast cancer [37]. While in the limited stage of SCLC, the expression of PARP1 was related to the progression free survival of tumor cells [38]. The COX regression analysis conducted by KJ Xie, et al. [39] indicated that PARP1 was an independent adverse prognostic factor of NSCLC patients. According to Kaplan-meier survival curve, the higher expression of PARP1, the lower survival rate of patients, which preliminary suggested that PARP1 might be an independent adverse prognostic factor of NSCLC. Consistently with the above result we found that PARP1 presented high expression in NSCLC, and the survival rate of patients was decreased accompanied by increased PARP1 expression. In summary, based on gene interaction network, the shortest paths method was adopted in NSCLC gene network to find a number of candidate oncogenes of NSCLC. We conducted the functional verification on PARP1, which provided convenience for the following discussion and study of the mechanisms related to the occurrence and development of NSCLC. We found the role of PARP1 in the migration and prognosis of NSCLC, laying foundations for the following clinical experiments, and providing theoretical basis for revealing the effects of PARP1 on NSCLC.

Based on the known NSCLC genes, this study found novel NSCLC gene PARP1 by using gene interaction network. and verified the promoting effect of PARP1 gene on the migration of non-small cell lung cancer.

## MATERIALS AND METHODS

### Network construction and shortest path algorithm

Data collection: taking “non-small cell lung cancer” as key word to retrieve and gain NSCLC-related gene from database of OMIM (Online Mendelian Inheritance in Man) (Rashbass, 1995), COSMIC (Catalogue of Somatic Mutations in Cancer) (Forbes et al., 2011) and GAD (The Genetic Association Database) (Becker, Barnes, Bright, & Wang, 2004).

Then the data acquired from STRING (version 9.0) (http://string.embl.de/) was used to build up initial weighted PPI network diagram in which all connected lines weight depended separately on confidence levels of interdependency between two genes.

We could make sure the shortest path of each pair of correlatively known gene of lung cancer and genes on the path by Dijkstra's algorithm: calculating optimal path of one node to all the other nodes, moreover, arraying these genes by Betweenness value that stood for internal nodes of certain gene contained all optimal paths found.

### Permutation test

No matter what kind of gene was included in our gene network and how the real node or gene was distributed, it was always easy to find the genes with high Betweenness value for some Betweenness value might sway by network basically structural models, yet these universal genes was not our wanted genes found from special genetic network. So we adopted permutation test to further screen NSCLC genes with optimal path in specific net.

From gene network, we randomly selected simulated genes involved with a number of NSCLC-associated genes and then calculated the shortest path within genes (a total of 500 simulations); after we counted once, the Betweenness value in stimulation was bigger than that of actual one. After 500 randomized trials, each gene would get a frequency that was defined as permutation FDR of shortest-path gene of which small value (FDR < 0.05) meant a significantly NSCLC-involved gene of the shortest path.

### Patients and clinical samples

There were serum and tumor biopsy samples of 75 patients with NSCLC, including 40 metastatic and 35 non-metastatic, staging by pathological diagnosis and International Association for the Study of Lung Cancer (IASLC) TNM Classification.

5 ml peripheral blood was drawn and put in vascular separator after admission. The serum was extracted within 2h and kept at −80°C.

Inclusive criteria: the experiment have met with medical ethics of Baoji Central Hospital approval; all patients without treatment before a month have sighed informed consent and got ECOG score ranging from 0-1 during hospitalization.

### Total RNA extraction

Total RNA from 8 cell lines, blood samples, 75 cancer tissues of NSCLC and the adjacent normal tissues were extracted in TRIZOL RNA extraction reagent (Invitrogen) by manual. The experiment was performed as follows: the tissues were added with 500 ul Trizol and 200 ul chloroform in order, after vibrating and standing, then were placed into centrifugal machine for 10 min, mixing with isopropanol and ethanol to discard supernatant by centrifugation; following that, the tissues were added with 20-50 ul DEPC-ddH2O to dissolving dissolve RNA using 20-50ul DEPC-ddH2O and preserved saving with absolute ethanol in refrigerator at −70°C.

### Reverse transcription PCR and QPCR

SuperScript III First-Strand Synthesis System kit (Invitrogen) was used to synthesize cDNA, with the detailed processes shown in brochure. The fluorescent primers were designed using primer premier 5.0 according to the sequence of genes. Real-time PCR measurements were performed on CFX96 (Bio-Rad). Each PCR reaction contained SYBR^®^ Premix Ex Taq (2x) 12.5 μl, 10 μmol/L upstream and downstream primers each 1 ul, cDNA template 2 μl, dH_2_O 10μl. The temperature process was 95°C for 30 sec followed by 40 cycles of amplification (95°C for 5 sec, 60°C for 30 sec, and 72°C for 30 sec). 2^−ΔΔt^ was adopted for date processing.

### NSCLC cell lines and culture of cells

NSCLC cell lines A549, H1975 and PC9, purchased from The Shanghai Institutes for Biological Sciences of the Chinese Academy of Sciences, were taken out and inoculated to RPMI-1640 culture solution complementary with 10% fetal bovine serum, and cultured at under 37°C with a humidity of 5%CO2 saturation, with liquid exchanging and passaging every 3-4 days.

### Construction of PARP1 gene silencing and overexpression of stable transgenic lines

Completed PARP1 cDNA would be acquired with primers (Table 1), and then we cloned completed PARP1 cDNA into pEGFP-N_2_vector to construct gene overexpression plasmid. As for the PARP1 gene silencing, shRNA and negative-control shRNA (shRNA-NC) of PRAP1 were designed according the sequence of PARP1 (NM_007415) from GenBank (Table 1). After annealing, shRNA of PRAP1 were ligated into pGPU6/GFP/Neo vector to build gene silencing recombinant plasmid. And then recombinant vectors were transfected into cell lines by liposome Lipofectamine 3000. Meanwhile, the blank vector transfection group was taken as the control group. After transfection for 2 days, there were part of green fluorescence cells which were converted to use 400 μg/ml G418 DMEM medium, screened for a month to attain gene silencing and overexpression stable transgenic lines, culture and cryopreserved in DMEM + 10% FBS + P.S. medium.

**Table 1:** Primers used for the detection and vector construction of PARP1

Usage	Forward	Reverse
qRT-PCR	GCAGAGTATGCCAAGTCCAACAG	ATCCACCTCATCGCCTTTTC
cDNA	GAAGATCTTCGCCATG GCGGAGTCTTCGGATAAGCT	CCGCTCGAGCGGCCA CAGGGAGGTCTTAAAAT
shRNA	CACCGGACCAAGTGTATGGTCAAGATTCA AGAGATCTTGACCA TACACTTGGTCCTTTTTTG	GATCCAAAAAAGGACCAA GTGTATGGTCAAGATCTCTTGA ATCTTGACCATACACTTGGTCC
shNC	CACCGTTCTCCGAACGTGT CACGTCAAGAGATTACGTGA CACGTTCGGAGAATTTTTTG	GATCCAAAAAATTCTCCGAACGTGTCACGT AATCTCTTGACGTGACACGTTCGGAGAAC

### Cell proliferation detection with MTT method

2 × 10^4^/ml cell suspension fluid was inoculated in 96-well plates, with each well having 3 parallel wells and about 100 μl fluid, and cultured in an incubator with 5% CO2 at 37°C to 24, 48, 72, 96 h, respectively. Following that, the plates were taken out, added with 50 μl MTT solution each well that was configured into 1 g/L with normal saline, and incubated at 37°C for 4 h to discard the supernatant fluid, then added with dimethylformamide (DMSO) (100 μl per well), shaken for 10 min to fully resolve MTT pyrolysis products. Optical density (OD) was measured by immunoassay analyzer at the wavelength of 570 nm, drawing cell growth curve.

### The scratch test and trans-well experiment for cell migration detection

For the scratch test, cells were plated at 2×10^5^ cells per well in a 6-well plate and grown overnight under standard conditions. After 12 h, the confluent monolayer was scratched manually with a plastic 200 μl pipette tip and after washing with PBS. Then the culture was continued in 2% serum at 37°C for 24 h. Cells that had migrated into the wound area were imaged using an inverted microscope at 100× magnification (Olympus, CKX41). The distance was measured using ImageJ2x.

Trans-well experiment was carried out manually. In brief, 1 × 10^5^ cells were plated onto 24-well cell chambers with a non-coated membrane (pore size: 8 μm, BD Biosciences). The cells were plated in medium without serum, and medium with 20% serum was used as a chemoattractant in the lower chamber. The cells were incubated at 37°C for 16 h, and cells that did not migrate through the pores were removed with a cotton swab. Cells on the lower surface of the membrane were fixed with 100% methanol for 5 min and stained with 0.1% crystal violet for 2 min (Sigma). Cells in 10 random fields of view at 100× magnification were counted and expressed as the average number of cells per field of view. Images were acquired using an inverted phase-contrast microscope at 100× magnification (Olympus, CKX41).

### Statistical analysis

All statistical analyses were performed using SPSS 21.0 statistical software. Measurement data were presented by mean ± standard deviation (AV ± SD). Unpaired Student's t-test for parametric data or Mann-Whitney rank sum test for nonparametric data were applied to analyze mean value between two groups. Chi-square tests were applied to determine count data expressed in n (%). In all cases, p < 0.05 was considered statistically significant.

## SUPPLEMENTARY MATERIALS TABLES







## Abbreviations

PARP1: poly (ADP-ribose) polymerase 1
NSCLC: non-small cell lung cancer
GO: gene ontology
KEGG: Kyoto encyclopedia of genes and genomes
OMIM: Online Mendelian Inheritance in Man
COSMIC: Catalogue of Somatic Mutations in Cancer
GAD: The Genetic Association Database
IASLC: International Association for the Study of Lung Cancer
