# Supplementary material for: An analysis of the gene interaction networks identifying the role of PARP1 in metastasis of non-small cell lung cancer
Source: Oncotarget. 2017 Aug 14;8(50):87263–75. doi: 10.18632/oncotarget.20256 (PMC5675631; doi:10.18632/oncotarget.20256)
Supplement: Supplementary file 1 [file oncotarget-08-87263-s001.pdf]

An analysis of the gene interaction networks identifying the role of PARP1 in metastasis of non-small cell lung cancer

SUPPLEMENTARY MATERIALS

Supplementary Table 1: The known gene of non-small cell lung cancer

See Supplementary File 1

Supplementary Table 2: Shortest path gene

See Supplementary File 2
