# Supplementary material for: An analysis of the gene interaction networks identifying the role of PARP1 in metastasis of non-small cell lung cancer
Source: Oncotarget. 2017 Aug 14;8(50):87263–75. doi: 10.18632/oncotarget.20256 (PMC5675631; doi:10.18632/oncotarget.20256)
Supplement: Supplementary file 2 [file oncotarget-08-87263-s002.docx]

**Supplementary Table 1: The known gene of non-small cell lung cancer**

| ABCB1 | Source | DisGeNET |
| --- | --- | --- |
| ABCC2 | Literature | ([Han et al., 2007](#_ENREF_28)) |
| ABCG2 | Literature | ([Han et al., 2007](#_ENREF_28)) |
| ACE | Literature | ([Yaren et al., 2008](#_ENREF_75)) |
| ADAMTS1 | Databases | DisGeNET |
| AGER | Databases | GAD |
| AKR1C1 | Databases | DisGeNET |
| AKT1 | Databases | COSMIC |
| AKT2 | Literature | ([Q. X. Li et al., 2013](#_ENREF_40)) |
| ALK | Databases | COSMIC,DisGeNET |
| APC | Databases | DisGeNET |
| APCDD1 | Literature | ([Ahn et al., 2012](#_ENREF_4)) |
| APEX1 | Literature | ([S. J. Kim et al., 2010](#_ENREF_35)) |
| AREG | Databases | DisGeNET |
| ATF3 | Databases | DisGeNET |
| ATM | Literature | ([Xiong et al., 2013](#_ENREF_71)) |
| ATR | Literature | ([Zienolddiny et al., 2006](#_ENREF_82)) |
| AURKA | Literature | ([Provencio et al., 2012](#_ENREF_53)) |
| AXL | Databases | DisGeNET |
| BAP1 | Databases | COSMIC |
| BCL2 | Literature | ([Masago et al., 2013](#_ENREF_46)) |
| BCL2L11 | Literature | ([Lee et al., 2014](#_ENREF_39)) |
| BIRC5 | Databases | DisGeNET |
| BRAF | Databases | COSMIC,OMIM |
| BRCA1 | Literature | ([Rosell et al., 2011](#_ENREF_58)) |
| BRCA2 | Literature | ([M. Kim et al., 2010](#_ENREF_34)) |
| CASP10 | Literature | ([Yoo et al., 2009](#_ENREF_76)) |
| CASP3 | Literature | ([Gu et al., 2012](#_ENREF_26)) |
| CASP7 | Literature | ([Yoo et al., 2009](#_ENREF_76)) |
| CASP8 | Databases | DisGeNET |
| CASP9 | Literature | ([Yoo et al., 2009](#_ENREF_76)) |
| CAT | Databases | DisGeNET |
| CCDC6 | Databases | COSMIC |
| CCL2 | Literature | ([Hildebrandt et al., 2010](#_ENREF_30)) |
| CCND1 | Literature | ([Gautschi et al., 2006](#_ENREF_23)) |
| CCNH | Literature | ([Ren et al., 2012](#_ENREF_56)) |
| CD74 | Databases | COSMIC,DisGeNET |
| CD9 | Databases | DisGeNET |
| CDA | Literature | ([Tibaldi et al., 2008](#_ENREF_65)) |
| CDH1 | Literature | ([G. Y. Wang et al., 2008](#_ENREF_68)) |
| CDH13 | Databases | DisGeNET |
| CDKN2A | Databases | DisGeNET |
| CHFR | Databases | DisGeNET |
| CHGA | Databases | DisGeNET |
| CHRNA3 | Literature | ([Z. J. Li, Bao, Xu, Bao, & Zhang, 2012](#_ENREF_42)) |
| CHRNA4 | Literature | ([Z. J. Li et al., 2012](#_ENREF_42)) |
| CHRNA5 | Literature | ([Z. J. Li et al., 2012](#_ENREF_42)) |
| CLPTM1L | Literature | ([Zienolddiny et al., 2009](#_ENREF_83)) |
| COMT | Literature | ([Y. J. Zhang et al., 2013](#_ENREF_80)) |
| CSF3 | Databases | DisGeNET |
| CTLA4 | Literature | ([Antczak, Pastuszak-Lewandoska, et al., 2013](#_ENREF_6)) |
| CYP17A1 | Literature | ([Y. J. Zhang et al., 2013](#_ENREF_80)) |
| CYP19A1 | Literature | ([Olivo-Marston et al., 2010](#_ENREF_51)) |
| CYP1A1 | Literature | ([C. Tan et al., 2011](#_ENREF_62)) |
| CYP1B1 | Literature | ([Ada et al., 2010](#_ENREF_1)) |
| CYP2C19 | Literature | ([Ada et al., 2010](#_ENREF_1)) |
| CYP2D6 | Literature | ([Ada et al., 2010](#_ENREF_1)) |
| CYP2E1 | Databases | DisGeNET |
| CYP3A4 | Literature | ([Islam et al., 2014](#_ENREF_31)) |
| CYP3A5 | Literature | ([Islam et al., 2014](#_ENREF_31)) |
| DAPK1 | Databases | DisGeNET |
| DCK | Literature | ([Ryu et al., 2012](#_ENREF_59)) |
| DDR2 | Literature | ([Nicos et al., 2014](#_ENREF_48)) |
| DHFR | Literature | ([Shimizu et al., 2012](#_ENREF_60)) |
| DUSP3 | Databases | DisGeNET |
| EGF | Literature | ([F. L. Tan et al., 2015](#_ENREF_63)) |
| EGFR | Databases | COSMIC,DisGeNET,OMIM |
| EML4 | Databases | COSMIC,DisGeNET |
| ENO2 | Databases | DisGeNET |
| EPHX1 | Literature | ([Gsur et al., 2003](#_ENREF_25)) |
| ERBB2 | Databases | COSMIC |
| ERCC1 | Databases | DisGeNET |
| ERCC2 | Literature | ([Yang & Xian, 2014](#_ENREF_74)) |
| ERCC4 | Literature | ([Lamba et al., 2014](#_ENREF_38)) |
| ERCC6 | Literature | ([Lamba et al., 2014](#_ENREF_38)) |
| EXO1 | Literature | ([Zienolddiny et al., 2006](#_ENREF_82)) |
| EZR | Databases | COSMIC |
| FAM38B | Literature | ([Ahn et al., 2012](#_ENREF_4)) |
| FAS | Literature | ([Choi et al., 2009](#_ENREF_15)) |
| FASLG | Databases | DisGeNET |
| FGF9 | Databases | DisGeNET |
| FGFR1 | Databases | DisGeNET |
| FGFR2 | Databases | COSMIC |
| FGFR4 | Literature | ([Fang, Tian, Zhou, Zhou, & Fang, 2013](#_ENREF_19)) |
| FHIT | Databases | DisGeNET |
| FOXM1 | Databases | DisGeNET |
| FOXO3 | Databases | DisGeNET |
| FZD4 | Literature | ([Coscio et al., 2014](#_ENREF_16)) |
| GCLC | Databases | DisGeNET |
| GGH | Literature | ([Adjei, Mandrekar, et al., 2010](#_ENREF_2)) |
| GNAS1 | Literature | ([Uzunoglu et al., 2013](#_ENREF_66)) |
| GSTM1 | Literature | ([H. Y. Zhang et al., 2014](#_ENREF_79)) |
| GSTM2 | Databases | DisGeNET |
| GSTM3 | Literature | ([Risch et al., 2001](#_ENREF_57)) |
| GSTP1 | Databases | DisGeNET |
| GSTT1 | Literature | ([H. Y. Zhang et al., 2014](#_ENREF_79)) |
| HIF1A | Literature | ([S. J. Kim et al., 2010](#_ENREF_35)) |
| HIP1 | Databases | COSMIC |
| HLA-A | Literature | ([Kotsakis et al., 2014](#_ENREF_36)) |
| HSPB1 | Literature | ([T. Xu et al., 2012](#_ENREF_73)) |
| IFNAR2 | Literature | ([Dai et al., 2012](#_ENREF_17)) |
| IFNG | Literature | ([Dai et al., 2012](#_ENREF_17)) |
| IGF1R | Literature | ([Reinmuth et al., 2014](#_ENREF_55)) |
| IGF2R | Literature | ([Kotsinas et al., 2008](#_ENREF_37)) |
| IL10 | Databases | DisGeNET |
| IL13 | Literature | ([Hildebrandt et al., 2010](#_ENREF_30)) |
| IL1A | Literature | ([Van Dyke et al., 2009](#_ENREF_67)) |
| IL1B | Literature | ([Van Dyke et al., 2009](#_ENREF_67)) |
| IL1RN | Literature | ([Van Dyke et al., 2009](#_ENREF_67)) |
| IL2 | Literature | ([Hildebrandt et al., 2010](#_ENREF_30)) |
| IL4 | Literature | ([Hildebrandt et al., 2010](#_ENREF_30)) |
| IL4R | Literature | ([Hildebrandt et al., 2010](#_ENREF_30)) |
| IL6 | Literature | ([Nie, Xue, Sun, Ning, & Zhao, 2014](#_ENREF_49)) |
| IL6R | Literature | ([Nie et al., 2014](#_ENREF_49)) |
| IL8 | Databases | DisGeNET |
| IRF1 | Databases | OMIM |
| KDM1A | Databases | DisGeNET |
| KDR | Databases | COSMIC |
| KIF5B | Databases | COSMIC,DisGeNET |
| KRAS | Databases | DisGeNET |
| LIG3 | Literature | ([Dong et al., 2012](#_ENREF_18)) |
| LIG4 | Literature | ([Jiang et al., 2014](#_ENREF_32)) |
| LRIG3 | Databases | COSMIC |
| LTA | Literature | ([Van Dyke et al., 2009](#_ENREF_67)) |
| MAP2K1 | Databases | COSMIC |
| MAP2K2 | Databases | COSMIC |
| MBD4 | Literature | ([Cho et al., 2011](#_ENREF_14)) |
| MDM2 | Databases | DisGeNET |
| MET | Databases | DisGeNET |
| MGMT | Literature | ([L. Wang, Liu, Zhang, Spitz, & Wei, 2006](#_ENREF_69)) |
| MIR126 | Databases | DisGeNET |
| MIR145 | Databases | DisGeNET |
| MLH1 | Literature | ([Antczak, Migdalska-Sek, et al., 2013](#_ENREF_5)) |
| MMP1 | Databases | DisGeNET |
| MMP11 | Databases | DisGeNET |
| MMP2 | Literature | ([Liu, Ping, Zu, & Sun, 2014](#_ENREF_43)) |
| MMP3 | Literature | ([Gonzalez-Arriaga et al., 2012](#_ENREF_24)) |
| MMP9 | Databases | DisGeNET |
| MOK | Databases | GAD |
| MPO | Literature | ([Chan, Lam, Fu, & Kwong, 2005](#_ENREF_10)) |
| MSH2 | Literature | ([Cheng et al., 2010](#_ENREF_13)) |
| MT3 | Databases | DisGeNET |
| MTHFR | Literature | ([X. Y. Li et al., 2014](#_ENREF_41)) |
| MUTYH | Literature | ([Qian et al., 2011](#_ENREF_54)) |
| NAPG | Literature | ([Ahn et al., 2012](#_ENREF_4)) |
| NAT1 | Literature | ([Wikman et al., 2001](#_ENREF_70)) |
| NAT2 | Literature | ([Wikman et al., 2001](#_ENREF_70)) |
| NBS1 | Literature | ([J. L. Xu et al., 2012](#_ENREF_72)) |
| NCOA3 | Databases | DisGeNET |
| NDRG1 | Databases | DisGeNET |
| NFE2L2 | Databases | COSMIC |
| NKX2-1 | Databases | COSMIC |
| NNAT | Databases | DisGeNET |
| NOS3 | Literature | ([Fujita et al., 2009](#_ENREF_22)) |
| NQO1 | Databases | DisGeNET |
| NRG1 | Databases | COSMIC |
| OGG1 | Literature | ([Park, Chen, Tockman, Elahi, & Lazarus, 2004](#_ENREF_52)) |
| PARP1 | Literature | ([Chang et al., 2009](#_ENREF_11)) |
| PLCH1 | Literature | ([Y. J. Zhang et al., 2013](#_ENREF_80)) |
| PIK3CA | Literature | OMIM |
| PPARG | Literature | ([Hildebrandt et al., 2010](#_ENREF_30)) |
| PPIA | Databases | DisGeNET |
| PRR13 | Databases | DisGeNET |
| PTEN | Literature | ([Q. X. Li et al., 2013](#_ENREF_40)) |
| PTGS2 | Literature | ([L. Liu et al., 2014](#_ENREF_44)) |
| PTHLH | Databases | DisGeNET |
| PXN | Databases | DisGeNET |
| PYCARD | Databases | DisGeNET |
| RAD51 | Literature | ([Nogueira et al., 2010](#_ENREF_50)) |
| RAF1 | \| Databases \| \| --- \| | DisGeNET |
| RAGE | Databases | GAD |
| RASSF1 | Databases | GAD,DisGeNET |
| RB1 | Databases | DisGeNET |
| RECK | Databases | DisGeNET |
| RET | Databases | COSMIC |
| ROS1 | Databases | COSMIC,DisGeNET |
| RRM1 | Databases | DisGeNET |
| SDC4 | Databases | COSMIC |
| SLC19A1 | Literature | ([Adjei, Salavaggione, et al., 2010](#_ENREF_3)) |
| SLC2A1 | Literature | ([S. J. Kim et al., 2010](#_ENREF_35)) |
| SLC34A2 | Databases | COSMIC |
| SMARCA4 | Databases | COSMIC |
| SOD2 | Databases | DisGeNET |
| SOX2 | Databases | COSMIC |
| SPP1 | Literature | ([Chen, Liu, Wu, Li, & Li, 2013](#_ENREF_12)) |
| STAT3 | Databases | DisGeNET |
| STK11 | Databases | COSMIC |
| SYP | Databases | DisGeNET |
| TERT | Databases | DisGeNET |
| TFG | Databases | COSMIC |
| TGFA | Databases | DisGeNET |
| TGFB1 | Literature | ([Teixeira et al., 2011](#_ENREF_64)) |
| TGFBR1 | Literature | ([Sun et al., 2011](#_ENREF_61)) |
| TGFBR2 | Literature | ([H. T. Zhang et al., 2004](#_ENREF_78)) |
| TMSB4X | Databases | GAD |
| TNF | Literature | ([Kaabachi et al., 2013](#_ENREF_33)) |
| TNFRSF1A | Literature | ([Kaabachi et al., 2013](#_ENREF_33)) |
| TNFRSF1B | Literature | ([Kaabachi et al., 2013](#_ENREF_33)) |
| TP53 | Databases | GAD,DisGeNET |
| TP53BP1 | Literature | ([Zheng et al., 2010](#_ENREF_81)) |
| TP63 | Literature | ([Lo Iacono et al., 2011](#_ENREF_45)) |
| TP73 | Literature | ([Lo Iacono et al., 2011](#_ENREF_45)) |
| TPM3 | Databases | COSMIC |
| TPR | Databases | COSMIC |
| TYMS | Literature | ([Yu et al., 2014](#_ENREF_77)) |
| UCHL1 | Databases | DisGeNET |
| UGT1A1 | Literature | ([Nakamura et al., 2011](#_ENREF_47)) |
| VDR | Literature | ([Heist et al., 2008](#_ENREF_29)) |
| VEGFA | Databases | GAD，DisGeNET |
| VIMP | Databases | DisGeNET |
| XPA | Literature | ([Feng et al., 2009](#_ENREF_20)) |
| XPD | Literature | ([Provencio et al., 2012](#_ENREF_53)) |
| XRCC1 | Databases | GAD |
| XRCC2 | Literature | ([Butkiewicz et al., 2012](#_ENREF_9)) |
| XRCC3 | Literature | ([Provencio et al., 2012](#_ENREF_53)) |
