# Supplementary material for: An analysis of the gene interaction networks identifying the role of PARP1 in metastasis of non-small cell lung cancer
Source: Oncotarget. 2017 Aug 14;8(50):87263–75. doi: 10.18632/oncotarget.20256 (PMC5675631; doi:10.18632/oncotarget.20256)
Supplement: Supplementary file 3 [file oncotarget-08-87263-s003.docx]

**Supplementary Table 2: Shortest path gene**

| ID | Name | betweenness | P-value |
| --- | --- | --- | --- |
| ENSG00000141510 | TP53 | 4187 | 0.009901 |
| ENSG00000100644 | HIF1A | 2383 | 0.009901 |
| ENSG00000177606 | JUN | 2280 | 0.039604 |
| ENSG00000132646 | PCNA | 1890 | 0.029703 |
| ENSG00000176890 | TYMS | 1359 | 0.019802 |
| ENSG00000127191 | TRAF2 | 1198 | 0.009901 |
| ENSG00000100030 | MAPK1 | 949 | 0.019802 |
| ENSG00000197442 | MAP3K5 | 914 | 0.009901 |
| ENSG00000106546 | AHR | 914 | 0.019802 |
| ENSG00000137752 | CASP1 | 900 | 0.009901 |
| ENSG00000143437 | ARNT | 899 | 0.019802 |
| ENSG00000132155 | RAF1 | 895 | 0.019802 |
| ENSG00000137275 | RIPK1 | 813 | 0.009901 |
| ENSG00000084676 | NCOA1 | 802 | 0.019802 |
| ENSG00000124181 | PLCG1 | 788 | 0.039604 |
| ENSG00000110092 | CCND1 | 770 | 0.039604 |
| ENSG00000140465 | CYP1A1 | 759 | 0.019802 |
| ENSG00000143799 | PARP1 | 757 | 0.009901 |
| ENSG00000134184 | GSTM1 | 746 | 0.009901 |
| ENSG00000150782 | IL18 | 709 | 0.009901 |
| ENSG00000198400 | NTRK1 | 689 | 0.029703 |
| ENSG00000169047 | IRS1 | 639 | 0.009901 |
| ENSG00000162434 | JAK1 | 613 | 0.019802 |
| ENSG00000166888 | STAT6 | 593 | 0.009901 |
| ENSG00000125538 | IL1B | 592 | 0.009901 |
| ENSG00000228716 | DHFR | 591 | 0.009901 |
| ENSG00000134259 | NGF | 589 | 0.009901 |
| ENSG00000095002 | MSH2 | 584 | 0.009901 |
| ENSG00000119318 | RAD23B | 578 | 0.009901 |
| ENSG00000073050 | XRCC1 | 570 | 0.009901 |
| ENSG00000112715 | VEGFA | 569 | 0.049505 |
| ENSG00000013275 | PSMC4 | 538 | 0.009901 |
| ENSG00000168040 | FADD | 473 | 0.009901 |
| ENSG00000186895 | FGF3 | 465 | 0.009901 |
| ENSG00000139618 | BRCA2 | 399 | 0.019802 |
| ENSG00000115590 | IL1R2 | 399 | 0.009901 |
| ENSG00000197971 | MBP | 398 | 0.009901 |
| ENSG00000136244 | IL6 | 398 | 0.049505 |
| ENSG00000142168 | SOD1 | 397 | 0.049505 |
| ENSG00000077238 | IL4R | 396 | 0.019802 |
| ENSG00000129187 | DCTD | 394 | 0.019802 |
| ENSG00000140505 | CYP1A2 | 389 | 0.009901 |
| ENSG00000136936 | XPA | 388 | 0.019802 |
| ENSG00000070501 | POLB | 375 | 0.009901 |
| ENSG00000101843 | PSMD10 | 357 | 0.009901 |
| ENSG00000134058 | CDK7 | 272 | 0.009901 |
| ENSG00000100985 | MMP9 | 216 | 0.019802 |
| ENSG00000102265 | TIMP1 | 216 | 0.009901 |
| ENSG00000137801 | THBS1 | 206 | 0.009901 |
| ENSG00000012061 | ERCC1 | 202 | 0.009901 |
| ENSG00000117971 | CHRNB4 | 201 | 0.009901 |
| ENSG00000149923 | PPP4C | 200 | 0.009901 |
| ENSG00000144381 | HSPD1 | 200 | 0.029703 |
| ENSG00000197386 | HTT | 200 | 0.039604 |
| ENSG00000126214 | KLC1 | 200 | 0.019802 |
| ENSG00000143190 | POU2F1 | 200 | 0.009901 |
| ENSG00000174827 | PDZK1 | 200 | 0.009901 |
| ENSG00000140564 | FURIN | 200 | 0.019802 |
| ENSG00000026508 | CD44 | 200 | 0.039604 |
| ENSG00000213366 | GSTM2 | 200 | 0.019802 |
| ENSG00000185379 | RAD51D | 200 | 0.009901 |
| ENSG00000135218 | CD36 | 200 | 0.019802 |
| ENSG00000075624 | ACTB | 200 | 0.039604 |
| ENSG00000129347 | KRI1 | 200 | 0.009901 |
| ENSG00000186111 | PIP5K1C | 200 | 0.009901 |
| ENSG00000136986 | DERL1 | 200 | 0.019802 |
| ENSG00000100983 | GSS | 200 | 0.009901 |
| ENSG00000073969 | NSF | 200 | 0.019802 |
| ENSG00000089289 | IGBP1 | 200 | 0.039604 |
| ENSG00000008294 | SPAG9 | 200 | 0.029703 |
| ENSG00000129514 | FOXA1 | 200 | 0.009901 |
| ENSG00000108384 | RAD51C | 200 | 0.009901 |
| ENSG00000105402 | NAPA | 200 | 0.019802 |
| ENSG00000172534 | HCFC1 | 200 | 0.029703 |
| ENSG00000121966 | CXCR4 | 200 | 0.039604 |
| ENSG00000111615 | KRR1 | 200 | 0.009901 |
| ENSG00000163501 | IHH | 199 | 0.029703 |
| ENSG00000165684 | SNAPC4 | 199 | 0.039604 |
| ENSG00000171094 | ALK | 199 | 0.039604 |
| ENSG00000104976 | SNAPC2 | 199 | 0.039604 |
| ENSG00000137076 | TLN1 | 198 | 0.029703 |
| ENSG00000137563 | GGH | 197 | 0.019802 |
| ENSG00000100823 | APEX1 | 195 | 0.039604 |
| ENSG00000100577 | GSTZ1 | 185 | 0.039604 |
| ENSG00000116044 | NFE2L2 | 119 | 0.039604 |
| ENSG00000113578 | FGF1 | 84 | 0.049505 |
| ENSG00000244067 | GSTA2 | 24 | 0.039604 |
| ENSG00000183087 | GAS6 | 21 | 0.029703 |
| ENSG00000102871 | TRADD | 18 | 0.009901 |
| ENSG00000134899 | ERCC5 | 14 | 0.009901 |
| ENSG00000255974 | CYP2A6 | 10 | 0.009901 |
| ENSG00000243955 | GSTA1 | 8 | 0.039604 |
| ENSG00000025423 | HSD17B6 | 8 | 0.009901 |
| ENSG00000171848 | RRM2 | 8 | 0.049505 |
| ENSG00000137074 | APTX | 4 | 0.029703 |
| ENSG00000244474 | UGT1A4 | 3 | 0.019802 |
| ENSG00000137364 | TPMT | 2 | 0.009901 |
| ENSG00000170310 | STX8 | 2 | 0.049505 |
| ENSG00000108786 | HSD17B1 | 2 | 0.009901 |
| ENSG00000157227 | MMP14 | 2 | 0.039604 |
| ENSG00000107731 | UNC5B | 1 | 0.009901 |
| ENSG00000111716 | LDHB | 1 | 0.029703 |
| ENSG00000104112 | SCG3 | 1 | 0.009901 |
